# *Ras* oncogene and *Hypoxia-inducible factor-1 alpha (hif-1*α*)* expression in the Amazon fish *Colossoma macropomum* (Cuvier, 1818) exposed to benzo[a]pyrene.

**DOI:** 10.1590/1678-4685-GMB-2016-0066

**Published:** 2017-05-08

**Authors:** Grazyelle Sebrenski da Silva, Luciana Mara Lopes Fé, Maria de Nazaré Paula da Silva, Vera Maria Fonseca de Almeida e Val

**Affiliations:** 1 Laboratory of Ecophysiology and Molecular Evolution (LEEM), Instituto Nacional de Pesquisas da Amazônia (INPA), Manaus, AM, Brazil; 2 Department of Morphology of the Institute of Biological Sciences (DM-ICB) Universidade Federal do Amazonas (UFAM), Manaus, AM, Brazil

**Keywords:** *Ras* oncogene, *hif-1*α, tambaqui, B[a]P, genotoxic damage

## Abstract

Benzo[a]pyrene (B[a]P) is a petroleum derivative capable of inducing cancer in human and animals. In this work, under laboratory conditions, we analyzed the responses of *Colossoma macropomum* to B[a]P acute exposure through intraperitoneal injection of four different B[a]P concentrations (4, 8, 16 and 32 μmol/kg) or corn oil (control group). We analyzed expression of the *ras* oncogene and the *Hypoxia-inducible factor-1 alpha* (*hif-1*α) gene using quantitative real-time PCR. Additionally, liver histopathological changes and genotoxic effects were evaluated through the comet assay. *Ras* oncogene was overexpressed in fish exposed to 4, 8 of 16 μmol/kg B[a]P, showing 4.96, 7.10 and 6.78-fold increases, respectively. Overexpression also occurred in *hif-1*α in fish injected with 4 and 8 μmol/kg B[a]P, showing 8.82 and 4.64-fold increases, respectively. Histopathological damage in fish liver was classified as irreparable in fish exposed to 8, 16 and 32 μmol/kg μM B[a]P. The genotoxic damage increased in fish injected with 8 and 16 μmol/kg in comparison with the control group. Acute exposure of B[a]P was capable to interrupt the expression of *ras* oncogene and *hif-1*α, and increase DNA breaks due to tissue damage.

## Introduction

Polycyclic aromatic hydrocarbons (PAHs) belong to a class of petroleum derivatives with high carcinogenic, mutagenic and genotoxic potential (Buhler and Williams, 1989, Vienneau *et al.*, 1995, Tsukatani *et al.*, 2003). PAHs are considered relevant threats to aquatic environments and are common contaminants in industrialized areas, mainly affecting inland and coastal water bodies, where organically enriched sediments or suspended particles may occur (Harris *et al.*, 1985, Meador *et al.*, 1995). PAHs contaminants can arise from natural sources, such as oil seeps, volcanoes, and forest fires, or from anthropogenic sources, as burning fuel, power generation, and oil spill (Latimer and Zheng, 2003).

Benzo[a]pyrene (B[a]P) is the most dangerous PAH, classified as Group 1 substance (IARC, International Agency for Research on Cancer, 2012). B[a]P is an immunosuppressive and pro-inflammatory agent, known as one of the most potent carcinogen (Pryor and Stone, 1993, Jaakkola and Jaakkola, 1997). To accomplish its carcinogenic action, B[a]P breaks down into reactive intermediates that covalently bind to DNA and cause a guanine (G)-thymine (T) transversion (Conney *et al.*, 1994).

The effects of B[a]P contamination have been studied in different groups of organisms such as fish (Padrós *et al.*, 2003), mussels (Tintos *et al.*, 2008), snails (Sanches-Arguello *et al.*, 2012), and mice (Gao *et al.*, 2011). Fish absorb PAHs from water via their body surface or gills and also by ingesting contaminated food or sediment (Varanasi *et al.*, 1989). In fish, exposure to PAHs results in the induction of enzymatic systems involved in the metabolism of xenobiotic compounds to detoxify the organism (Buhler and Williams, 1989). Additionally, histological alterations also occur in the liver of fish exposed to B[a]P. Oliveira-Ribeiro *et al.* (2007) described degenerative lesions, nuclear pleomorphism, pre-neoplastic proliferative conditions and necrosis as typical lesions in the fish liver. Due to strikingly similar histopathological features between fish and human tumors, fish have been used as models in cancer research (Lam *et al.*, 2006).

Recently, gene expression profiling has attracted researchers as a means of comparing the molecular features of tumors among different vertebrate species (Grabher and Thomas, 2006). For instance, rainbow trout (*Oncorhynchus mykiss*) has many advantages as a human carcinogenesis study model. These characteristics include the effects of PAHs (Bailey *et al.*, 1987, 1996) and the responses of some genes, such as *ras* oncogenes (Rotchell *et al.*, 2001).

Ras proteins that play a central role in cell growth signaling cascades. To date, several *ras* genes are characterized in fish, and have a high degree of similarity with mammalian nucleotide and deduced amino acid sequences. In fact, some species of fish have been used as models to understand *ras* genes behavior and their homology with human genes (Rotchell *et al.*, 2001). Goldfish (*Carassius auratus*) was the first fish to have its *ras* gene studied by Nemoto *et al.* (1986). Later, other fish species were investigated such as rainbow trout (Mangold *et al.*, 1991), zebrafish (*Danio rerio*) (Cheng *et al.*, 1997) and medaka (*Oryzias latipes*) (Rotchell *et al.*, 2001).

Another gene related to cancer development is *Hypoxia-inducible factor-1 alpha* (*hif-1*α), which produces the protein (HIF-1α) that is the major regulator of oxygen-dependent gene expression (Maxwell *et al.*, 1997, Maxwell, 2005, Rytkönen *et al.*, 2008, Fraga *et al.*, 2009). The levels of *hif-1*α expression are associated with tumorigenesis and angiogenesis (Zhong *et al.*, 1999). Although *hif-1*α has been mostly associated with hypoxic responses in fish, tumor cell hypoxia is also a well-studied system (Geng *et al.*, 2014). Tumor investigation is now seen as an integral part of the basic biological approach to elucidate the common mechanisms of cancer at different phylogenetic levels (Van Beneden *et al.*, 1990).

In Brazil, tambaqui (*Colossoma macropomum*) is one of the largest freshwater fish species. This species belongs to the Characidae family and is endemic to the Amazon basin. It is found mainly in rivers and in floodplain lakes (Várzea Lakes) (Marcuschi *et al.*, 2010). In the Amazon basin, tambaqui is one of the most important commercial fish (Val and Honczaryk, 1995). It also presents many characteristics of an appropriate bioindicator species for biomonitoring programs (Salazar-Lugo *et al.*, 2011).

Herein, we report the acute effects of B[a]P injections in tambaqui on *ras* oncogene expression as well as on *hif-1*α gene expression. We used fish liver to investigate gene expression and histopathological damages, and peripheral blood to investigate the genotoxic effects of B[a]P.

## Materials and Methods

### Animals

Juveniles of *C. macropomum* (24.76 ± 5.45 g; 10.50 ± 0.64 cm) were purchased from a local fish farm nearby Manaus city (Santo Antônio Farm), Amazon State (Brazil). Fish were transported to the Laboratory of Ecophysiology and Molecular Evolution at the National Institute for Amazonian Research (LEEM - INPA). Fish were held indoors in fish tanks supplied with recirculating aerated INPA’s groundwater (in μmol L^-1^ [Na^+^], 43; [K^+^], 10; [Ca^2+^], 9; [Mg^2+^], 4; [Cl^-^], 31; [Cu^2+^], 7.0 μgL^-1^; hardness = 1.33 CaCO_3_ mgL ^-1^; pH = 6.80); and fed once a day with commercial feed containing 36% protein. Fish were monitored daily during the acclimation period (7 days).

After the first acclimation period, 15 animals were transferred to six plastic tanks (70 L capacity) containing water with constant aeration. Each fish was weighed and measured, to calculate the amount of pollutant to be intraperitoneally injected. Then, fish were allowed to acclimate in these tanks for at least seven days before beginning the tests. Physicochemical parameters were measured over the course of the experiment using a digital oxygen meter YSI (Yellow Springs Instruments, USA) model 55/12-155 for temperature (26.05 ± 0.23 °C) and dissolved oxygen (7.45 ± 0.21 mg/L). A digital pH-meter UltraBASIC UB-10 (Denver 156 Instrument) was used to measure the pH (5.75 ± 0.16).

After one week of acclimation, feeding was suspended and fish starved for 24 h before starting the acute experiment (96 h). Each fish received an injection volume in accordance with the weight. Independently of treatment, the volume of the vehicle to be injected (corn oil) was calculated using a weight ratio (0.01 mL/g). We followed the recommended protocols described in the Brazilian Guides of Animal Care and Use, and as required by the Ethics Committee on Animal Use of the National Institute for Amazonian Research (CEUA – INPA) (Protocol Number 011/2013). Sample size was the minimum required for each method, observing the literature and CONCEA recommendations. We used five treatments for the whole experiment (n = 10 for each treatment): i) control group, injected with corn oil; and the other four groups injected with a solution containing corn oil as vehicle and four concentrations of B[a]P as follows: ii) 4 μmol/kg B[a]P, iii) 8 μmol/kg B[a]P, iv) 16 μmol/kg B[a]P, and v) 32 μmol/kg B[a]P. Before receiving the injection, animals were anesthetized on ice, and after the injection they were kept in the tanks for 96 h. After this period, blood was sampled with a heparinized syringe from the caudal vein, and then fish were euthanized through cerebral concussion followed by severing of the anterior spinal cord. The fish liver was dissected and one portion was snap-frozen in liquid nitrogen and stored at -80 °C. The other portion was fixed in Alfac solution as described below for histopathology analysis through light microscopy.

### Histopathology analysis of liver

Six liver samples from each treatment were immediately fixed in Alfac solution (70% ethanol, 5% glacial acetic acid, and 4% formaldehyde) for 16 h, dehydrated in a graded series of ethanol, and embedded in Paraplast Plus^®^ (Sigma). Sections of 5 μm were obtained, stained with hematoxylin/eosin and observed at 40 objective magnification in a light microscope.

Histopathological alterations index (HAI) scores were semi-quantitatively calculated using the method described by Poleksic and Mitrovic-Tutundic (1994). Indices based on the severity of lesions were used to asses liver tissue changes: I = Σ I + 10 Σ II + 100 Σ III, where stages I, II, and III correspond to the degree of the lesion. The final Index is described as follows: normal liver function (I = 0–10), mild to moderate damage (I = 11–20), moderate to the severe damage (I = 21–50), severe damage (I = 51–100), and irreparable damage (I > 100).

### Comet assay in erythrocytes

We quantified the DNA damage in erythrocytes using the comet assay as described by Singh *et al.* (1988), and modified by Silva *et al.* (2000). Two comet microscope slides for ten fish from each treatment were prepared with standard melting agarose (1.5% normal melting agarose prepared in phosphate-buffer saline [PBS]) and dried overnight. Five microliters of whole fish blood were mixed with 0.75% low melting point agarose at 5% ratio (Gibco, Brazil) at 37 °C and immediately poured on pre-covered slides. Each slide was covered with a coverslip until the agarose solidified and then gently removed. Slides were placed in a lysis solution consisting of high salts and detergents (2.5M NaCl, 100 mM EDTA, 10 mM Tris, pH 10–10.5; 1% Triton X-100 and 10% DMSO). Before electrophoresis, the slides were incubated for 20 min in alkaline electrophoresis buffer (300 mM NaOH and 1 mM EDTA, pH > 13) to produce single-stranded DNA. After alkaline-unwinding, the single-stranded DNA was electrophoresed in the gels in a dark place under alkaline conditions for 20 min at 300 mA and 25 V at 4 °C to produce the comets. After electrophoresis, the slides were rinsed with a suitable buffer (0.4 M Tris buffer, pH 7.5) to neutralize the alkalis in the gels. Finally, DNA was revealed with silver solution staining (5% sodium carbonate, 0.1% ammonia nitrate, 0.1% silver nitrate, 0.25% tungstosilicic acid and 0.15% formaldehyde). Slides were examined using a light microscope (Leica DM 500) at 40 objective magnification. Randomly selected cells (100 cells from each of two replicate slides) were analyzed for each animal. We used the tail sizes to score the comet assay into five classes (from undamaged (zero) to maximum damage (four)). An overall score was obtained by addition of all cell scores from completely undamaged (sum zero) to maximum damage (sum 400), according to Kobayashi *et al.* (1995).

### Isolation of total RNA and cDNA synthesis

Isolation of total RNA from four tambaqui liver from each treatment group followed the TRIzol^®^ reagent protocol (Invitrogen) according to the manufacturer’s instructions. Contaminating genomic DNA was removed using DNase I (Invitrogen).

First strand cDNA was reverse-transcribed from the total RNA using RevertAid H Minus First Strand cDNA Synthesis kit (Fermentas), and following the manufacturer’s instructions. Enzymatic treatment with reverse transcriptase (MMLV Reverse Transcriptase) (200 U/μL, USB) was first done, and then mixed in a 0.2 mL microtube with approximately 25 μg RNA, 1 μL of Oligo(dT)_18_ primer (1 μg), 1.0 μL dNTP mix (10 mM), MMLV buffer 5X, and deionized water for a 20 μL final volume. This solution was incubated at 37 °C for 1 h for conversion and at 70 °C for 10 min to inactivate the enzyme.

### Determination of *ras* and *hif-1*α sequences

Degenerate primers were designed based on the conserved regions of *28S* (Vásquez, 2009), *ef-1*α*, ras* and *hif-1*α genes described in the NCBI database for other fish species. We used these primers to obtain partial fragments of tambaqui *ras* and *hif-1*α cDNAs. PCR amplification was performed using PCR Master Mix (Promega). All PCR products were sequenced with ABI PRISM^®^ BigDye^TM^ Terminator Cycle Sequencing Ready Reaction kit (Applied Biosystems) and run on an ABI 3130XL automatic DNA sequencer (Applied Biosystems). The sequences were analyzed using the BLAST program from NCBI and then used to design the specific primers for *Colossoma macropomum* q-PCR: *ras*, *hif-1*α (target primers), *28S* and *ef-1*α (reference primers) shown in Table 1.

**Table 1:** Details of primers designed for reference genes (*28S* and *ef-1*α) and the two target genes (*ras* and *hif-1*α).

*Gene Symbol*	Primer sequence (5’-3”) forward/reverse	Length (bp)	Amplicon length (bp)	Tm	Eff (%)^a^
*28S-F* b	CGGGTTCGTTTGCGTTAC	18	150	54.5	98.19
*28S-R* b	AAAGGGTGTCGGGTTCAGAT	20	150	56.3	98.19
*EF-1*α*F*	GTTGGTGAGTTTGAGGCTGG	20	78	60.7	99.09
*EF-1*α*R*	CACTCCCAGGGTGAAAGC	18	78	60.9	99.09
*Ras-F*	CCAGTACATGAGGACAGGAG	20	134	60.3	99.31
*Ras-R*	CAAGCACCATTGGCACATCG	20	134	60.3	99.31
*HIF-1*α*F*	CTTCTGAGCTCTGATGAGGC	20	98	60.1	105.24
*HIF-1*α*R*	GAAAGCACCATCAGGAAGCC	20	98	61.2	105.24

a Primer efficiency

b Vásquez (2009)

### Quantitative real-time PCR

A ViiA^TM^ 7 Dx system (Applied Biosystems) was used as a platform to quantify gene transcripts by real-time PCR. We analyzed four samples *C. macropomum* liver for each treatment. The reaction mixture consisted of 1 μL of cDNA as template (added in triplicate to the wells of a 96-well thin-wall PCR plate), 1 μL of each primer (concentration of *ras*: 2 pmol; *hif-1*α: 2 pmol, *28S*: 2.5 pmol and *ef-1*α: 1.5 pmol), 2 μL of nuclease-free water 192 (Ambion, Life Technologies) and 5 μL of SYBR Green PCR Master Mix (Applied Biosystems) in a total volume of 10 μL. The PCR protocol was: 2 min at 50 °C and 95 °C for 10 min, followed by 40 cycles of 95 °C for 15 s and 60 °C for 1 min (annealing temperature of all primers). By melting curve analysis the presence of a single product-specific melting temperature was confirmed: *28S* (slope -3.36/ R^2^ 0.99), *ef-1*α (slope -.3.34/ R^2^ 0.99), *ras* (slope -3.33/ R^2^ 0.97) and *hif-1*α (slope -3.20/ R^2^ 0.99). Amplification efficiency for each primer set was calculated from a serial dilution curve obtained from a pool of experimental samples (1000 to 1 ng cDNA concentration; n = 4). All primer pairs showed high PCR efficiency (between 98–105%). Serial dilutions of a cDNA standard were amplified in each run to determine amplification efficiency according to Pfaffl (2001).

### Statistical analysis

All data are reported as mean ± SEM (standard errors of means). Gene expression, histopathology and comet assay data were analyzed by one-way analysis of variance (ANOVA) to assess differences between the treatment and control groups. When the data violated the premises of one-way ANOVA test, a Kruskal-Wallis one-way analysis of variance or rank test was applied. Statistical significance was accepted at the level of p < 0.05. Statistical analysis was performed using the statistical program Sigma Stat 3.5.

## Results

**Figure 1:**
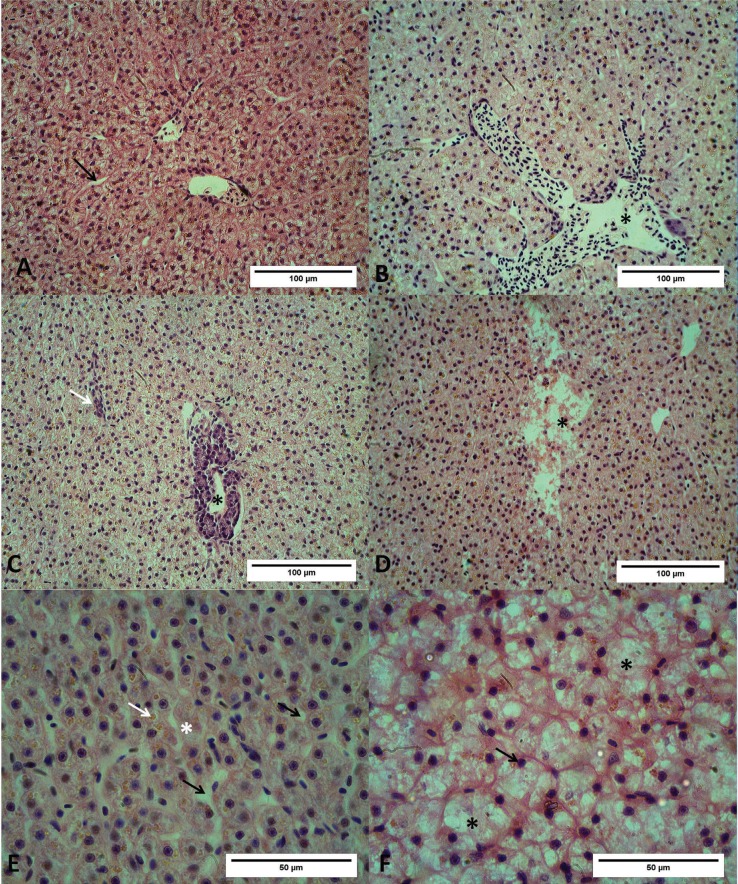
*C. macropomum* liver exposed to corn oil (control group) or doses of B[a]P. (A) Normal liver, hepatocytes are organized in one or two layers surrounded by sinusoids (black arrows). (B) Normal liver parenchyma, highlighting a blood vessel with red blood cells (asterisk). (C) Image of liver exposed to 8 μmol/kg B[a]P evidencing a hepatopancreas (asterisk) and sinusoid obstruction (white arrow). (D) Image of fish liver exposed to 8 μmol/kg B[a]P, showing necrotic area (asterisk). (E) Image of liver exposed to 16 μmol/kg B[a]P showing some hepatocytes without nucleus (white asterisk), sinusoidal dilatation (black arrows) and hemosiderin (white arrow). (F) Image of vacuolated hepatocytes of fish exposed to 32 μmol/kg B[a]P; cytoplasm degeneration (black asterisks) and pyknotic nuclei (black arrow) are evident. Slides were stained with hematoxylin and eosin.

The liver of *C. macropomum* has a similar morphological structure as that of other fish species, as observed in liver slides from the group control. This group exhibited mild to moderate damage, according to the histopathological analyses classification (Figure 1A) (Poleksic and Mitrovic-Tutundic, 1994). A healthy liver presents polygonal hepatocytes with very prominent central nuclei. Hepatocytes are arranged into two-cell thick cords surrounded by sinusoidal epithelial cells (Figure 1B) (Genten *et al.*, 2009). Damage in fish groups exposed to 8, 16, and 32 μmol/kg of BaP were irreparable, according to the HAI classification (Poleksic and Mitrovic-Tutundic, 1994).

We observed cytoplasm vacuolization, cell hypertrophy, nuclei hypertrophy, and parenchyma disorganization in all treatments with B[a]P (Figure 1). Severe cytoplasm vacuolization occurred in the liver of fish exposed to 32 μmol/kg B[a]P: small vacuoles appeared in the cellular cytoplasm and subsequently fused to form a larger vacuole. As a consequence, the cell vacuoles forced cytoplasm and nuclei to the periphery of the cell. We also observed infiltration of leucocytes as an inflammatory sign in all exposed fish. Altered hepatocytes presented cytoplasm degeneration accompanied by an alteration in shape and size, losing their characteristic polyhedral shape and frequently showing hypertrophy (Figure 1F). Plasmatic membrane rupture was common in fish exposed to 8, 16, and 32 μmol/kg B[a]P. These groups also presented focal necrosis in almost all animals (Figure 1D).

**Figure 2:**
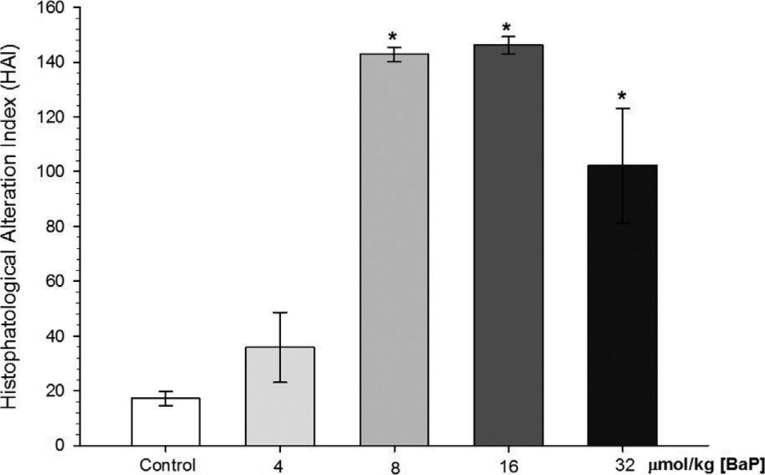
Histopathological alteration index (HAI) of *C. macropomum* liver after exposure to different concentrations of B[a]P. Indexes are in accordance with Poleksic and Mitrovic-Tutundic (1994). *Indicates significant differences compared to control group (corn oil) (p < 0.05). Kruskal-Wallis test was used.

Observing the HAI index described by Poleksic and Mitrovic-Tutundic (1994), the occurrence of liver damage was evident in fish exposed to the higher B[a]P concentrations; 8, 16, and 32 μmol/kg B[a]P (HAI = 142.80 ± 2.6, 146.16 ± 3.09, and 102.16 ± 20.89, respectively) (Figure 2).

**Figure 3:**
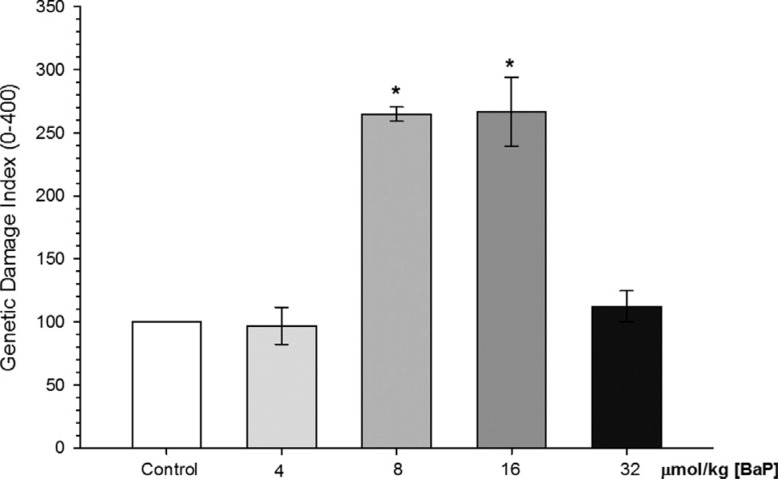
Genetic damage index (GDI) of erythrocytes of *C. macropomum* after 96 h of injection of different concentrations of B[a]P. *Indicates significant differences compared to control group (corn oil) (p < 0.05). Kruskal-Wallis test was used.

Genetic damage as measured through the comet assay was induced in the acute experiment (96 h) with B[a]P. Exposure to B[a]P caused a significant genotoxic effect in *C. macropomum* exposed to 8 (GDI = 264 ± 5.66) and 16 μmol/kg (GDI = 266 ± 27.31), in comparison with control. No difference was found in fish exposed to 32 μmol/kg B[a]P (112.35 ± 12.16) compared to the control group (Figure 3).

**Figure 4:**
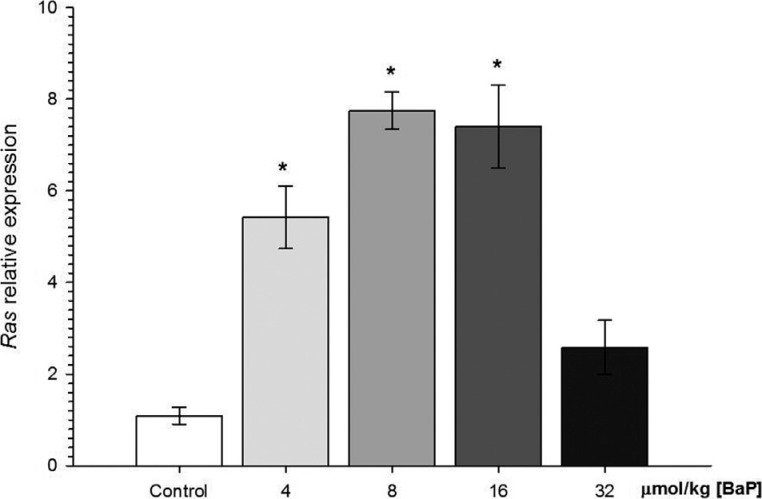
Relative expression of the oncogene *ras* in liver of *C. macropomum* after 96 h of injection of different concentrations of B[a]P. *Indicates significant difference in comparison to control group (p < 0.05). Kruskal-Wallis test was used.

An increase was observed in the expression of *ras* oncogene in *C. macropomum* exposed for 96 h to 4, 8 and 16 μmol/kg B[a]P in comparison to the control (Figure 4). *Ras* oncogene was overexpressed 4.96-fold in fish exposed to 4 μmol/kg of B[a]P, 7.10-fold in fish exposed to 8 μmol/kg and 6.78-fold in fish exposed to 16 μmol/kg of B[a]P. There was no difference in the expression of *ras* in the 32 μmol/kg B[a]P group compared to the control group.

**Figure 5:**
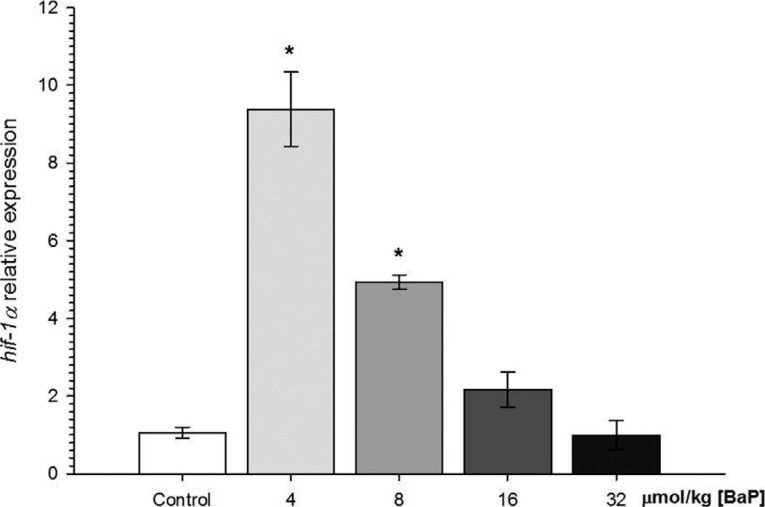
Relative expression of gene *hif-1*α gene in *C. macropomum* after 96 h of injection of different concentrations of B[a]P. *Indicates significant difference in comparison to control group (corn oil) (p < 0.05). Kruskal-Wallis test was used.

The expression of *hif-1*α increased approximately 8.82-fold in fish injected with 4 μmol/kg B[a]P and approximately 4.64-fold in fish injected with 8 μmol/kg B[a]P in comparison with the control group (Figure 5). However in the higher concentration of B[a]P (16 and 32 μmol/kg), the expression of *hif-1*α was similar with the control group.

## Discussion

Histopathological liver damage caused by exposure to B[a]P and petroleum derivatives are largely described in the literature (Malmstrom *et al.*, 2004, Costa *et al.*, 2010, Agamy, 2012, Moller *et al.*, 2014). Liver is one of the most important organs to be addressed, since it is responsible for the detoxification process in the organism, and it is the primary organ in the biotransformation of organic xenobiotics (Health, 1995, Hinton *et al.*, 2001, Rojo-Nieto *et al.*, 2014).

Many investigations have shown that focal, multifocal and diffuse vacuolar degeneration of hepatocytes are the result of fish exposure to a variety of different carcinogenic agents (Couch, 1975, Mathur, 1975, Stehr *et al.*, 1998, Nero *et al.*, 2006, Stentiford *et al.*, 2014). We also detected cell hypertrophy, followed by loss of polyhedral shape, inflammatory focus with leucocytes infiltration, cytoplasmic degeneration, and parenchyma disorganization in these fish. Agamy (2012) described hepatocytes with marked nuclear enlargement and moderate cellular enlargement, along with an alteration in shape and size, losing their typical polyhedric shape and frequently presenting hypertrophy in the liver of juvenile rabbit fish exposed to the oil water accommodated fraction (WAF). Malmstrom *et al.* (2004) also verified a massive infiltration of inflammatory cells in rainbow trout (*Oncorhynchus mykiss*) and cytoplasmic vacuolization in flounder (*Platichthys flesus*) intraperitoneally injected with B[a]P. Multifocal inflammatory lesions in the liver were recognized in other two teleosts, Atlantic cod (*Gadus morhua*) and flounder (*Platichthys flesus*), caged for three months on contaminated sediments in a Norwegian fjord (Husoy *et al.*, 1996). In the present work, liver histopathology of *C. macropomum* exposed to different concentrations of B[a]P revealed an increase in tissue injuries in a dose-dependent way. In all treatments, we could observe cellular vacuolization, as was also observed in the liver of juvenile rabbit fish (*Siganus canaliculatus*) exposed to a WAF of light Arabian crude oil (Agamy, 2012). Liver hepatic parenchyma disorganization appears to be correlated with PAH exposure (Rojo-Nieto *et al.*, 2014). In our study, the lesions observed in *C. macropomum* liver were associated with PAH injection, indicating the extreme toxic potential of this compound to aquatic animals. These lesions were more evident in focal necrosis of hepatocytes, observed in most of the *C. macropomum* livers after treatment with B[a]P. Agamy (2012), studying rabbit fish (*Siganus canaliculatus*) exposed to dispersed oil for six days, found hepatocyte necrosis and cellular swelling on fish liver, which became larger with increased time of exposure. In another study with eelpout (*Zoarces viviparus*) collected in differently polluted areas, necrosis and degeneration were observed and the cellular structure was no longer maintained, with eosinophilic cytoplasm elements and free pyknotic nuclei being visible within the liver sections (Fricke *et al.*, 2014). As observed in the present work, Abdel-Moneim *et al.* (2012) also described foci of local hepatic tissue necrosis characterized by entirely destroyed hepatic tubules and, in most cases, no remaining hepatic cellular structure. In our study, we observed that some fish also contained lysed hepatocyte remnants. Thus, we suggest that acute exposure to this pollutant induced liver damage that impairs normal liver function in these animals.

The analysis of DNA damage in aquatic organisms has been considered a highly suitable method for evaluating genotoxicity caused by contamination of environments. In general, the comet assay method is considered advantageous because it detects and quantifies the genotoxic impact without requiring a detailed knowledge of the identity or the physical/chemical properties of the contaminants (Frenzilli *et al.*, 2009). Numerous studies evidenced DNA strand break using the comet assay in different animal models (Lemiere *et al.*, 2005, Lacaze *et al.*, 2010, Michel *et al.*, 2013). In this study, the comet assay indicated DNA damage in *C. macropomum* blood cells of fish injected with 8 and 16 μmol/kg μM B[a]P in comparison with the control. There was no difference among groups injected with corn oil, 4 and 32 μmol/kg B[a]P. This can be explained by the release of new erythrocyte cells due to the high concentration of the pollutant, which puts a high cost on the individual’s defense system. Also, mechanisms of DNA repair in erythrocytes may have been activated. Our results are similar to those of Jeong *et al.* (2015). These authors examined the degree of DNA damage in beakfish (*Oplegnathus fasciatus*) caused by three fractions (aliphatic hydrocarbons, aromatic hydrocarbons, and polar compounds) of sediment organic extracts taken from Taean (Korea). The DNA damage level was the highest in cells exposed to 1.00 mg/g dry weight (dw) followed by the 1.09 mg/g dw and 0.72 mg/g dw to PAH. Studying DNA damage in gill and liver of carp and rainbow trout, Kim and Hyun (2006) observed similar results. In their study, the level of damage was very low during the initial 24 h of exposure to B[a]P and increased dramatically during the next 24 h, and then gradually decreased until 96 h. Curtis *et al.* (2011) observed the same result in rainbow trout exposed to B[a]P, where damage to blood cell DNA increased in fish fed a diet contaminated with B[a]P after 14 and 28 days compared to controls. In our study, the DNA damage in fish injected with an intermediary concentration of B[a]P was higher. Future investigation concerning DNA repair mechanisms should help to understand the decrease in DNA damage in fish injected with higher amounts of B[a]P.

Another way to evaluate the effects of certain pollutants as carcinogenic inducers is through the alteration in gene expressions or mutations (Ostrander and Rotchell, 2005). The oncogene *ras* is considered one of the most important genes involved in carcinogenesis. The characterization of this gene in several fish species and the presence of *ras* mutations have already been described in fish populations inhabiting hydrocarbon contaminated areas and following experimental exposure to specific contaminants (Nogueira *et al.*, 2006). In the present study, the *ras* oncogene was found overexpressed in livers of fish treated with 4, 8, and 16 μmol/kg B[a]P. When comparing these data with DNA damage in erythrocytes, significant differences in DNA damage were apparent only at concentrations of 8 and 16 μmol/kg of B[a]P. Our data suggest that the oncogene *ras* is expressed even with exposure to low concentrations of the contaminant, and DNA damage is more significant when animals are subjected to higher concentrations. However, in fish injected with the 8 and 16 μmol/kg concentrations, DNA damage and *ras* oncogene expression responded similarly to the presence of the contaminant. Nogueira *et al.* (2010), studying *Dicentrarchus labrax* and *Liza aurata* in a contaminated coastal lagoon polluted by PAH, observed no differences in the expression levels of *ras* oncogene among fish from different sites. Similar results were found in *Anguilla anguilla* exposed to 0.1 and 0.3 μM B[a]P, where the analysis of *ras* oncogene revealed no differences in levels of expression between control and exposed fish (Nogueira *et al.*, 2006). In another study with mussels (*Mytilus galloprovincialis*) collected in sites with different levels of petrochemical contamination along the NW coast of Portugal, the expression of *ras* oncogene in digestive gland and gonads was decreased in PAH-contaminated animals. These authors also found similar results in fish exposed to 100% WAF (Lima *et al.*, 2008). According to Rotchell *et al.* (2001), the pattern and incidence of *ras* oncogene mutations in environmentally induced tumors also appear to be species-specific in fish. Tumors were not observed in tissue liver analyses in this study due to the short exposure period, but the described histopathology characteristics may certainly lead to tumor formation as an inflammatory focus with longer exposure to the pollutant B[a]P (Grivennikov *et al.*, 2010). Additionally, overexpression of the oncogene *ras* observed herein is among the mechanisms that implicate carcinogenesis (Nogueira *et al.*, 2006).

Another gene related with cancer is *hif-1*α, which has been identified as a key regulator of angiogenesis, inflammation and anaerobic metabolism (Dehne and Brune, 2009). Importantly in the past few years, *hif-1*α has been implicated in the development of a range of liver pathologies, such as liver fibrosis, activation of the immune system, hepatocellular carcinoma, and others, in humans, as well as in rodents (Nath and Szabo, 2012, Semenza, 2012). In humans, many studies have emphasized the metastasis process in solid tumors induced by the expression of *hif-1*α (Schweiki *et al.*, 1992, Melstrom *et al.*, 2011). Most hypoxia studies have been focused on mammalian systems (Taylor and Sivakumar, 2005). However, hypoxia is a common phenomenon also for fish. In fish, the majority of studies describe the expression of *hif-1*α in hypoxic environmental condition, but not considering the combined effects of hypoxia and pollution (Terova *et al.*, 2008).

In the present study, hypoxia was not a challenge to *C. macropomum*, but the challenge was the contaminant (B[a]P). The highest expression of *hif-1*α occurred at the lowest concentration of B[a]P, suggesting that the hepatocytes were capable of activating the transcription of this gene, helping to maintain the cell survival machinery, as evidenced by the literature, which correlates *hif-1*α with cell proliferation and survival (Siddiq *et al.*, 2007). In fish exposed to the highest concentration of B[a]P, the cellular machinery was already compromised by cell damage, and the tissue was not efficient in controlling gene expression to keep levels of *hif-1*α high, since the normal functioning of the liver was impaired by necrosis. Yu *et al.* (2008) suggested that the application of xenobiotics such as B[a]P to hypoxia-stressed fish induces the increase in HIF-1-mediated transcription, particularly in xenobiotic-metabolizing organs such as the liver. The orange-spotted grouper (*Epinephelus coioides*) was examined upon single and combined exposures to hypoxia and B[a]P. The responses for four hypoxia-responsive (HIF-1-mediated) genes [*igfbp* (*insulin-like growth factor binding protein*), *epo* (*erythropoietin*), *ldh-a* (*lactate dehydrogenase* an isoform) and *vegf* (*vascular endothelial growth factor*)] in fish liver tissues were monitored at four different time intervals using real-time qPCR. The authors showed that B[a]P did not alter the expression of these four genes throughout the course of the exposure to normoxic conditions. Although when combined with hypoxia, the pollutant caused the activation of these genes at some concentrations. Under hypoxia, these genes were very responsive. In fact, the *hif-1*α gene encodes a transcription factor controling more than 100 genes, including genes responsible for immune processes and inflammation of cells (Yu *et al.*, 2008).

As far as we know, this is the first study that analyses in combination the responses of the *ras* and *hif-1*α genes in a Neotropical freshwater fish (*C. macropomum*) under acute exposure to B[a]P at normoxic conditions. Both gene expression and comet assay analyses showed full bell-shaped dose-response results. We observed an increase in gene expression and erythrocyte DNA damage in fish exposed to 4, 8 and 16 μmol/kg B[a]P, and a decrease of these responses in fish exposed to the highest concentration (32 μmol/kg B[a]P). Bosveld *et al.* (2002) showed response to PAHs in their study with ethoxyresorufin dealkylase (EROD) activity in the H4IIE rat hepatoma in an *in vitro* bioassay. These authors observed that a category of compounds such as indeno[1,2,3-cd]pyrene (IP), benz[a]anthracene (BaA), B[a]P, chrysene (Chr), and benzo[k]fluoranthene (BkF), induces strong reactions, showing full bell-shaped dose–response relationships over a wide dose range and with a strong increase in EROD activity. Lu *et al.* (2009) also observed bell-shaped dose-response in their study with *Carassius auratus* exposed to the PAH indeno[1,2,3-cd]pyrene via intraperitoneal injection at dosages of 0.1, 1, 2, 5 and 10 (or 8) mg/kg. The EROD activity resulted in a decreased fold-induction at the highest dosage of indeno[1,2,3-cd]pyrene (10 mg/kg), as well as glutathione S-transferase, which showed the same behavior. Bell-shaped curves have been reported for various *in vitro* and *in vivo* systems after exposure to PAHs (Kennedy *et al.*, 1996, Delesclue *et al.*, 1997).

The majority of the works addressing *ras* oncogenes are done in humans (Maertens and Cichowski, 2014). The studies with *hif-1*α also describe the expression of this gene in human solid tumors, and in metastasis (Fraga *et al.*, 2009). In fish species, many authors study this gene as a marker for environmental hypoxia condition without a pollutant (Rissanen *et al.*, 2006, Rimoldi *et al.*, 2012). Ongoing studies in our laboratory combining pollutants and hypoxia exposure and exposure to different climate scenarios should further help to respond how these genes respond to synergistic effects.

## Conclusion

Amazonian fish have proven to be versatile as bioindicators of environmental pollution, using both toxicology and genotoxicity markers. In the present work, we observed that the species *C. macropomum* is sensible to B[a]P under acute exposure. However, further studies are necessary to better understand the behavior of the *ras* and *hif-1*α genes under the effects of contaminants. The exposure of this species to this pollutant for a longer time and along with other environmental stresses is under development. This work contributed with essential data to further understand the role of these genes in the cell machinery, especially when a contaminant is involved. The mechanisms related in the overexpression of *ras* and *hif-1*α genes on the intermediary concentration of B[a]P needs further study.
